# Outcomes of definitive radiotherapy vs. laryngectomy followed by adjuvant radiotherapy in patients with locally advanced laryngeal squamous cell carcinoma: real-world experience in a referral cancer center

**DOI:** 10.1186/s13014-024-02565-9

**Published:** 2024-12-18

**Authors:** Ali Kazemian, Ebrahim Esmati, Reza Ghalehtaki, Borna Farazmand, Nima Mousavi-Darzikolaee, Reyhaneh Bayani, Mahdieh Razmkhah, Maryam Taherioun, Niloufar Saeedi, Farrokh Heidari, Kaveh Zakeri

**Affiliations:** 1 Department of Radiation Oncology, Cancer Institute, IKHC, Tehran University of Medical Sciences, Tehran, Iran; 2 Radiation Oncology Research Center (RORC), Cancer Research Institute, Tehran University of Medical Sciences, Tehran, Iran; 3 Division of Laryngology, Department of Otorhinolaryngology and Head and Neck Surgery, Amir A’lam Hospital, Tehran University of Medical Sciences, Tehran, Iran; 4 Department of Otorhinolaryngology and Head and Neck Surgery, Amir A’lam Hospital, Tehran University of Medical Sciences, Tehran, Iran; 5 Department of Radiation Oncology, Memorial Sloan Kettering Cancer Center, New York, NY USA

**Keywords:** Laryngeal neoplasms, Head and Neck Neoplasms, Radiotherapy, Radiotherapy, Adjuvant, Drug therapy, Induction chemotherapy, Consolidation chemotherapy, Maintenance chemotherapy

## Abstract

**Background:**

Laryngeal cancer is a common head and neck cancer. Surgical treatment can impair patients’ voice and swallowing function, making definitive radiotherapy a viable alternative for locally advanced cases.

**Methods:**

To compare the outcomes of definitive versus adjuvant radiotherapy in patients with primary locally advanced laryngeal cancer, we retrospectively evaluated consecutive patients treated from 2007 to 2020. We assessed and compared the median and 3-year overall survival (OS), disease-free survival (DFS), distant metastasis control (DMC), and local recurrence-free survival (LRC) in all patients and in T4 patients exclusively.

**Results:**

One hundred patients were studied, including definitive (*N* = 64) and adjuvant (*N* = 36) radiotherapy. The median follow-up was 29 months. Overall, the median OS in the definitive vs. adjuvant group was 100 months (95%CI = 46.5-153.5) vs. not reached, respectively (log-rank *P* = 0.506). The median DFS in the definitive vs. adjuvant group was 20 months (95%CI = 7.7–32.3) vs. not reached, respectively (log-rank *P* = 0.148). Three-year OS and DFS rates in all patients were 64% (95%CI: 48–78) vs. 75% (95%CI: 55–95) and 43% (95%CI:29–57) vs. 61% (95%CI: 41–81) in the definitive vs. adjuvant groups, respectively. Among T4 patients, the median OS in the definitive RT group vs. adjuvant group was not reached vs. 48 (95%CI = 0-105.3), respectively (log-rank *P* = 0.788). The median DFS in the definitive RT group vs. adjuvant group was 12 months (95%CI = 9.34–14.65) vs. 36 months (95%CI = 4.4–67.5), respectively (log-rank *P* = 0.868). Three-year OS and DFS rates were 71% (95%CI: 42–100) vs. 75% (95%CI: 50–100) and 40% (95%CI:21–79) vs. 56% (95%CI: 25–87) in the definitive vs. adjuvant groups, respectively.

**Conclusions:**

Our analysis suggests that definitive radiotherapy in laryngeal cancer does not lead to a poorer outcome than total laryngectomy followed by adjuvant radiotherapy. In T4 patients, our findings should reassure clinicians and patients about the viability of definitive radiotherapy as a treatment approach.

## Background

Laryngeal cancer is a common head and neck cancer, with an overall incidence of 2.76 and a prevalence of 14.33 cases/year per 100,000 population worldwide, and showing an increasing trend in recent decades [1]. The incidence of laryngeal cancer in Iran is comparable to the global incidence, with an age-standardized rate of 2.7 cases/year per 100,000 men [2]. The incidence peaks after the age of 65, with a 5-fold higher incidence in males, which may be attributable to the close association of laryngeal cancer with tobacco use and smoking [3]. An overall mortality rate of 1.66 deaths/year per 100,000 population has been reported for laryngeal cancer. However, there has been a 5% decrease in mortality over the past decades as a result of detection at earlier stages or advances in treatment strategies [1].

Laryngeal cancer is highly curable in its early stages, with a success rate of approximately 80–100% [4]. However, in locally advanced diseases with large tumors and/or involvement of cervical lymph nodes, the control rate is lower, ranging from 40 to 70%. In these cases, total laryngectomy followed by adjuvant radiation and chemotherapy is suggested by many as the pivotal treatment strategy, especially in T4a cases [5, 6]. However, total laryngectomy (TL) has a significant drawback as it can impair patient’s voice and swallowing function, impacting their communication ability and quality of life (QOL) [7]. Organ-preservation strategies, including sequential chemoradiotherapy or definitive chemoradiotherapy (definitive), have been suggested as alternative strategies for these patients [8, 9]. Some experts consider primary non-surgical treatment, especially for younger patients with moderately advanced tumors, because of acceptable loco-regional control and larynx preservation [10]. Nevertheless, the optimal therapeutic approach in each stage remains a subject of debate and poses challenges due to insufficient evidence supporting the selection of the best strategy [11]. Considering the ongoing investigations in this area and the need for further studies in a clinical setting to determine the safety and efficacy, the present study aimed to compare the patients’ outcomes after definitive compared to upfront total laryngectomy followed by adjuvant (chemo) radiation (adjuvant).

## Methods

### Study design and participants

In this retrospective cohort study, we aimed to compare the effect of definitive versus adjuvant radiotherapy in patients diagnosed with primary locally advanced laryngeal cancer who were referred to the radiation oncology ward of Cancer Institute, affiliated with the Tehran University of Medical Sciences in 2007–2020. Diagnosis of laryngeal cancer was confirmed by pathology reports, and those with squamous cell carcinoma (SCC) who completed their radiotherapy course were included. Individuals with recurrent laryngeal cancer, distant metastases, or a history of previous radiation to the head and neck were excluded from the study. The study received approval from the institutional review board (code: 99-1-248-47949) and ethics committee (code: IR.TUMS.IKHC.REC.1399.102).

### Institution protocols

All patients were evaluated, treated, and followed by our institutional protocols. Patients who have a dysfunctional larynx that makes normal breathing and swallowing impossible should undergo total laryngectomy (TL). Patients who have a remarkable thyroid or cricoid cartilage invasion are potential candidates for TL unless they refuse it or are medically unfit. Patients with fixed true vocal cords whose tumors are relatively large, without a desirable voice that is not expected to get much better after radiotherapy, are encouraged to undergo TL. Initiating induction chemotherapy and deciding based on tumor response is not a routine practice in our institution.

### Pretreatment evaluation

Pretreatment evaluation included a comprehensive medical history, physical examination, and video laryngoscopy. In addition, magnetic resonance imaging (MRI) or computed tomography (CT) scan of the neck with intravenous contrast was performed to assess the extension of the primary tumor and the presence of lymphadenopathy. If there was any suspicion of an involved neck lymph node, a neck ultrasound with or without biopsy was ordered. CT and MRI criteria for lymphadenopathy included: short-axis diameter ≥ 10 mm, cluster of ≥ 3 lymph nodes with borderline size, necrosis, and extranodal extension (ENE).

In allocating stage and contouring for radiotherapy (RT), all clinically suspicious nodes that weren’t biopsied were considered malignant unless proven otherwise. All patients underwent a thoracic CT scan with contrast to evaluate distant metastasis. The Airway was assessed before treatment to determine the necessity for tracheostomy.

The tumor characteristics, including T, N, M, and overall staging based on AJCC 7th edition were recorded in the study checklist, based on the pathology report and clinical workups in the adjuvant and definitive groups, respectively.

### Treatment protocol

All patients in both groups were treated with 3-dimensional conformal radiation therapy (3D-CRT). Our department’s simulation and treatment planning protocol for 3DCRT was followed that is described here. For the delineation of gross tumor volume (GTV) and the clinical target volume (CTV), we used the same protocol as the RTOG 91 − 11 trial [12], taking into account the findings of laryngoscopy, MRI and/or CT scan, and physical examination. Patients were immobilized for CT simulation and treatment with a thermoplastic mask, with an extended neck and suppressed shoulder position. All RT treatments were delivered in 2 Gy per fraction sessions unless stated otherwise. We primarily used a photon energy of 6MV. Surgery was performed by routine techniques for primary or salvage laryngectomy described elsewhere [13].

Treatment details specific to each group are described below.

#### Group 1: definitive radiotherapy

Patients with locally advanced laryngeal SCC (T3, T4, and/or positive nodes) who were treated with definitive radiotherapy with or without chemotherapy were included in this group. The chemotherapy protocols included both induction and concurrent regimens. For induction chemotherapy, the indications were inducing a tumor response to improve breathing and swallowing or initiating treatment to compromise the effect of long waiting list of radiotherapy. We used either the TPF regimen (docetaxel 75mg/m^2^ day one, cisplatin 25mg/m^2^ days 1–3, and bolus 5-fluorouracil [5FU] 425mg/m^2^ on days 1–4) with granulocyte colony stimulating factor support or PF regimen (same as TPF without docetaxel) every 3 weeks for 1–3 cycles. The concurrent chemotherapy was indicated for patients younger than 70 years with good performance. The chemoradiotherapy regimen included cisplatin 30-35mg/m^2^ weekly or 100mg/m^2^ every 3 weeks. Patients who were not fit to receive cisplatin, due to underlying renal or liver diseases, heart failure, intolerance to high volumes of fluid needed for cisplatin injection, neuropathy, or ototoxicity, received a loading dose of 400mg/m^2^ cetuximab one week prior to initiation of RT and 250mg/m^2^ weekly concurrently with RT.

The total planned dose of definitive radiotherapy was 70 Gy. GTV was defined as gross primary tumor and involved lymph nodes (as was described in the pretreatment section). In patients who received induction chemotherapy, post-chemotherapy residual disease was considered GTV. CTV70 was defined as GTV plus 0–5 mm margin based on certainty and planning target volume (PTV)-70 was defined as CTV70 plus a 5 mm margin. CTV60 included CTV70 with a margin, whole larynx, and involved lymph node levels. After induction chemotherapy, CTV60 included primary GTV with 1 cm margin, whole larynx, and involved lymph node levels before chemotherapy. CTV46 included CTV60 plus elective lymph nodes. To delineate elective CTV of 46 Gy, in all laryngeal subsites, bilateral cervical lymph node levels II-IV were included. For supraglottic lesions invading the oral tongue, level Ib was also included in the CTV46. Elective level VI was also included in tumors approaching or involving the anterior commissure, subglottis or contralateral side of the larynx.

#### Group 2: upfront laryngectomy followed by adjuvant radiotherapy

In our department, patients who underwent TL were indicated for adjuvant radiotherapy if they had any of the following: clinical T3, pT3/T4, pN2 or higher, extra-nodal extension (ENE), lymphovascular or perineural invasion, close or positive surgical margin(s), and/or subglottic invasion more than 1 cm.

The dose for adjuvant RT was 60 Gy overall, except for patients with positive margin(s) and/or ENE who received 66 Gy, including a 6 Gy-boost for the positive margin site(s) or the node(s) with ENE. In cases with gross residual disease (R2 resection), the dose was boosted up to 70 Gy. CTV60 encompassed the operative bed, the surgical scar, the stoma, and the node-positive neck. Elective node contouring and radiation dose (46 Gy) were the same as the definitive group.

Patients with ENE and/or positive margin(s) were indicated for concurrent chemotherapy. Additionally, the patients younger than 60 years who were deemed high-risk for recurrence based on multiple indications for postoperative RT also received concurrent chemotherapy.

### Patient assessment during and after radiotherapy

We assessed patients based on our routine clinic protocol every week during RT to identify any adverse effects of treatment including fatigue, mucositis/pharyngitis, dermatitis, nausea/vomiting, and dysphagia/odynophagia. Medical history, physical examination, and laboratory tests were performed in each appointment. The chemotherapy-related toxicity was evaluated by serum tests, reflected as leukopenia, neutropenia, anemia, and/or thrombocytopenia. Patients who received cisplatin were also evaluated for renal toxicity, neuropathy, ototoxicity, hypomagnesemia and hypocalcemia, and nausea/vomiting. Those who received cetuximab were assessed for acneiform skin eruptions, mucositis, and hypomagnesemia. Toxicity results are not reported in the study.

We recorded the date of completion of radiotherapy and chemotherapy (before, during, and after RT) in the study checklist. Patients who received at least 66 Gy in the definitive setting and 60 Gy in the postoperative RT setting were considered fully treated. The sufficiency of concurrent chemotherapy in the definitive group was determined based on receiving at least 200mg/m^2^ cumulative dose of cisplatin or 1900mg/m^2^ of cetuximab. For the adjuvant group, receiving at least 150mg/m^2^ of cisplatin in patients who were deemed to require concurrent chemotherapy was considered sufficient. Of course, many patients in adjuvant didn’t require concurrent systemic therapy based on indications.

Post-treatment follow-up included evaluation of recurrence and metastasis to estimate overall survival (OS), disease-free survival (DFS), distant metastasis control (DMC), and locoregional-free survival (LRC). Recurrence was defined as the emergence of a new mass in imaging or laryngoscopy exam, confirmed by biopsy. Metastasis was considered as the involvement of any distant organs or lymph nodes. A second primary tumor was defined as the emergence of a new tumor in a distinct location with a different pathology or the same pathology but occurring after 5 years of the primary treatment.

### Statistical analyses

The collected data was analyzed using R Version 4.3.2. Descriptive analyses were performed, including frequency for categorical variables and mean ± standard deviation (SD) or median for numeric variables, based on the normal distribution of the selected variable. The normal age distribution across the groups was confirmed using the one-sample Kolmogorov-Smirnov test, while RT duration did not follow a normal distribution. To compare numeric variables, independent samples t-test was used for age, and the Mann-Whitney U test was used for RT duration, based on the distribution of the data across the study groups. Log-rank test was employed for evaluation of the patients’ outcomes, including overall OS, EFS, MFS, and LRC. A significance level of *P* < 0.05 was considered statistically significant for all analytical tests.

## Results

### Patient characteristics

A total of 100 patients were included in this study; 64 patients in the definitive group and 36 in the adjuvant group. The mean ± standard deviation (SD) of patients’ age was as follows: 61.72 ± 11.79 years in the definitive group and 58.17 ± 8.90 years in the adjuvant group (*P* = 0.093). In the definitive and adjuvant groups, 95.3% and 97.2% were male, respectively (*P* = 0.545). Among patients with T4 stage, the reasons for not undergoing surgery were as follows: Patient refusal 7 (46.7%), unfitness 1 (6.7%), unresectability 2 (13.3%), and selected T4a cases suitable for laryngeal preservation 5 (33.3%).

As shown in Table 1, comparing tumor characteristics between the two study groups revealed a lower frequency of T4 tumors in the definitive group (28.1% vs. 41.7%; *P* = 0.167).

Only 17% of the definitive radiotherapy group received induction chemotherapy; this rate was 11% in the adjuvant group. Concurrent systemic therapy was indicated in 55 patients in the definitive group (85.9%), and 18 patients (50%) in the adjuvant group. In total, 17 (26.6%) patients in the definitive group and 7 (19.4%) patients in the adjuvant group didn’t receive sufficient concurrent systemic therapy or received no systemic treatment, although indicated. So, chemotherapy compliance was 59.4% in the definitive group (38 patients out of 55) and 30.6% in the adjuvant group (11 patients out of 18).

**Table 1:** Comparison of patient and tumor characteristics between the two study groups

Variable	Categories	Definitive	Adjuvant	*P*-value^*^
T stage	T3	46 (71.9)	21 (58.3)	0.112
	T4a	13 (21.3)	14 (40)	
	T4b	2 (3.3)	0 (0)	
N stage	0	31 (48.4)	16 (44.4)	0.884
	1	10 (15.6)	5 (13.9)	
	2	18 (28.1)	13 (36.1)	
	3	5 (7.8)	2 (5.6)	
Stage	III	32 (50)	13 (36.1)	0.300
	IVa	27 (42.2)	21 (58.3)	
	IVb	5 (7.8)	2 (5.6)	
Completion of RT	Yes	58 (90.6)	31 (86.1)	0.498
	No	6 (9.4)	5 (13.9)	
Chemotherapy before RT	Yes	11 (17.2)	4 (11.1)	0.414
	No	53 (82.8)	32 (88.9)	
Concurrent systemic therapy	Yes	55 (85.9)	18 (50)	< 0.001
	No	9 (14.1)	18 (50)	
Regimen of concurrent systemic therapy	Cisplatin, weekly	33 (60)	16 (88.9)	0.108
	Cisplatin, triweekly	20 (36.4)	2 (11.1)	
	Erbitux	2 (3.6)	0	
Systemic therapy compliance (when indicated)	Yes	38 (69.1)	7 (38.8)	< 0.001
	No	17 (30.9)	11 (61.2)	

^*^The result of the chi-square test

### Outcomes in the two groups overall

**Fig. 1:**
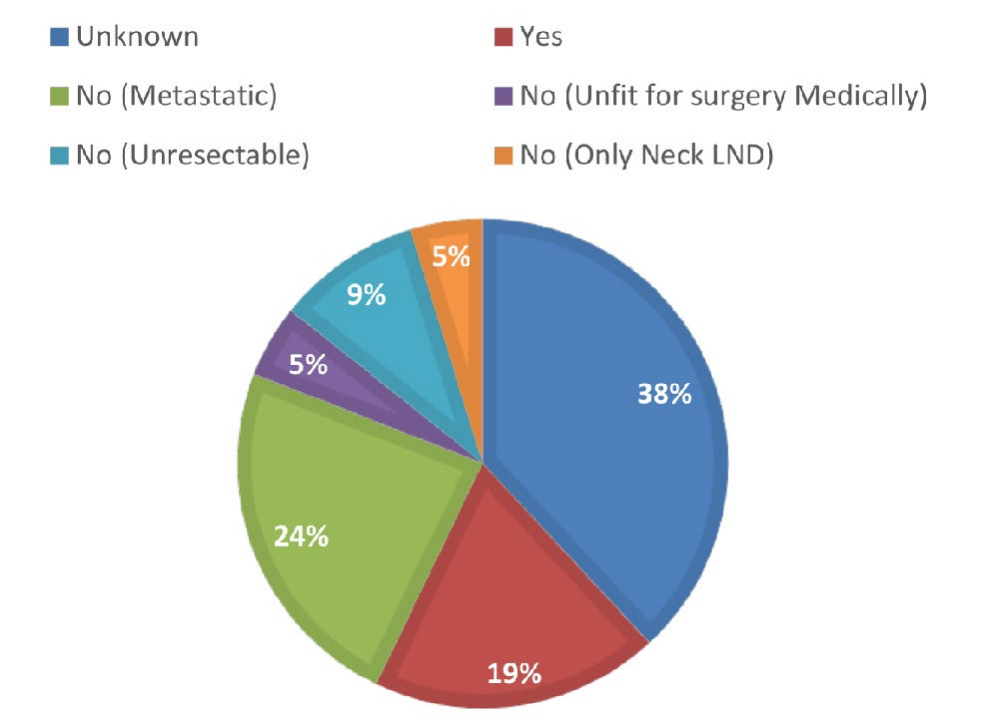
The rate of salvage laryngectomy or reasons of not undergoing surgery at the time of recurrence in the definitive group

During a median follow-up period of 29 months (reverse Kaplan-Meier, 95% confidence interval [CI]: 17.1–40.9), recurrence occurred in 20 patients, distant metastasis in 14 patients, and death in 29 patients. Among the 21 patients who experienced recurrence in the definitive group, the surgery rate or reasons for not undergoing salvage laryngectomy has been shown in Fig. 1.

**Fig. 2:**
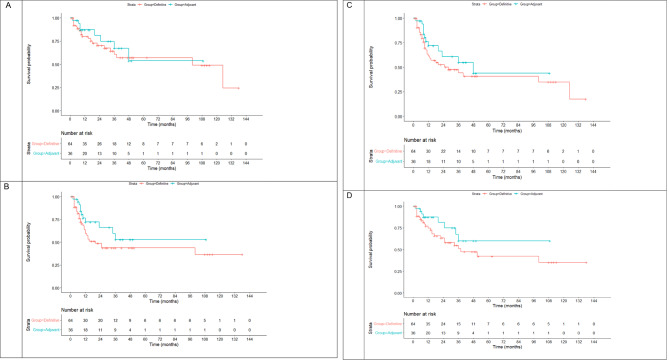
Kaplan-Meier curves for overall survival (**A**), disease-free survival (**B**), locoregional recurrence-free survival (**C**), and distant metastasis control (**D**) in all patients

The median OS in the definitive vs. adjuvant group was 100 months (95%CI = 46.5-153.5) vs. not reached, respectively (log-rank *P* = 0.506). The median DFS in the definitive vs. adjuvant group was 20 months (95%CI = 7.7–32.3) vs. not reached, respectively (log-rank *P* = 0.148). The median LRC in the definitive vs. adjuvant group was 28 months (95%CI = 3.7–52.3) vs. 48 months (95%CI = 10.3–85.6), respectively (log-rank *P* = 0.250). The median DMC in the definitive vs. adjuvant group was 38 (95%CI = 11.9–64.1) vs. not reached, respectively (log-rank *P* = 0.150). The corresponding KM curves are shown in Fig. 2 (A-D). The 3-year rates are shown in Table 2.

**Table 2:** 3-year overall, disease-free, locoregional control, and distant metastasis-control rates based on all patients

	3-year OS	3-year DFS	3-year LRC	3-year DMC
All Definitive	64 (48–78)	43 (29–57)	48 (34–62)	54 (38–70)
All Adjuvant	75 (55–95)	61 (41–81)	63 (43–83)	68 (46–90)
*P*	0.265	0.138	0.185	0.235

### Outcomes in T4 disease

**Fig. 3:**
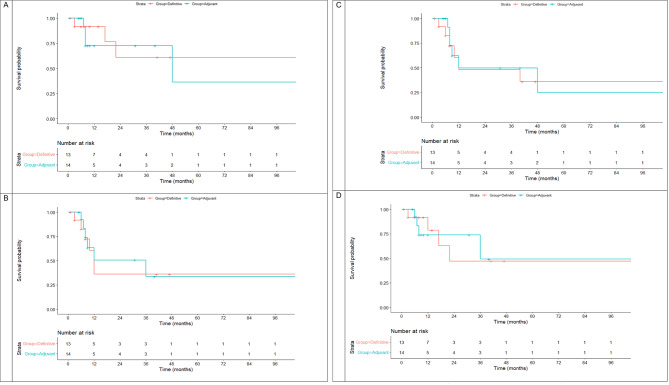
Kaplan-Meier curves for overall survival (**A**), disease-free survival (**B**), locoregional recurrence-free survival (**C**), and distant metastasis control (**D**) in T4 patients

The median OS in the definitive vs. adjuvant group was not reached vs. 48 (95%CI = 0-105.3) months, respectively (log-rank *P* = 0.788) (Fig. 3). The median DFS in the definitive vs. adjuvant group was 12 (95%CI = 9.34–14.65) vs. 36 (95%CI = 4.4–67.5) months, respectively (log-rank *P* = 0.868). The median LRC in the definitive vs. adjuvant group was 12 (95%CI = 0-52.7) vs. 12 (95%CI = 0-45.5), respectively (log-rank *P* = 0.250). The median DMC in the definitive vs. adjuvant group was 59 (95%CI = 24.5–95.1) vs. 64 (95%CI = 29.4–99.3) months, respectively (log-rank *P* = 0.150). The corresponding KM curves are shown in Fig. 2 (A-D).

Table 3 shows the 3-year rates of OS, DFS, LRC, and DMC in T4a patients. Although the 3-year OS was not different between the definitive and adjuvant groups, the DMC rates showed a remarkable but non-significant difference, and the LRC rates showed a modest difference between the two groups.

## Discussion

The comparative analysis conducted in this study aimed to evaluate the outcomes of patients with primary locally advanced laryngeal squamous cell carcinoma (SCC) who underwent definitive chemoradiotherapy or surgery followed by adjuvant radiation therapy. Here, we will discuss the key findings and their implications. First, we should note that In the definitive group of our cohort, 86% received concurrent chemotherapy, while only 17% of patients received induction chemotherapy. The present study did not find a statistically significant difference in overall survival (OS) between the definitive and adjuvant groups. The median OS was not reached in the definitive group, while it was 48 months in the adjuvant group. However, the 95% confidence intervals for OS were wide, suggesting that the study may have been underpowered to detect an actual difference in survival rates. Similarly, there was no significant difference in disease-free survival (DFS) between the two groups. The median DFS was 12 months in the definitive group and 36 months in the adjuvant group. Again, the confidence intervals for DFS were wide. The dissociation between DFS and OS is explained by the successful salvage treatment for the definitive group, the phenomenon that is rarely available after total laryngectomy followed by adjuvant radiotherapy.

**Table 3:** 3-year overall, disease-free, locoregional failure-free, and distant metastasis-control rates in T4a patients

	3-year OS	3-year DFS	3-year LRC	3-year DMC
T4a Definitive	71 (42–100)	40 (11–69)	50 (21–79)	48 (15–81)
T4a Adjuvant	75 (50–100)	56 (25–87)	56 (25–87)	75 (50–100)
*P*-value	0.390	0.306	0.384	0.180

The efforts to reach a non-surgical laryngeal preservation in locally advanced laryngeal cancers back to 1970s. Primordial studies failed to suggest a similar local control and survival to surgery. Eventually, the successful Veterans’ Administration Laryngeal Cancer Study Group (VALCSG) trial showed that patient selection through induction chemotherapy (IC) using cisplatin and fluorouracil (PF) could optimally select suitable candidates for laryngeal preservation with radiotherapy [14]. Later on, the RTOG 91 − 11 trial showed that in patients with a functional larynx, concurrent chemoradiotherapy and IC followed by RT yields similar results [12]. After the establishment of IC followed by RT in responders to preserve the larynx, the GORTEC 2000-01 phase III trial showed the superiority ability in laryngeal preservation of a triplet induction regimen (TPF, docetaxel plus PF) over the conventional PF regimen (70% vs. 60%) followed by RT alone in those with complete response at the primary site or partial response and recovered normal larynx mobility without a difference in 3-year OS (both 60%) [15]. In the same path, in the TREMPLIN phase II trial, following the IC using TPF regimen in those with at least 50% response, bioradiotherapy using cetuximab showed similar results to chemoradiotherapy using cisplatin. The 18-month laryngeal function preservation (82–87%) and 3-year OS (73–75%) was the highest in this trial compared to its ancestors [16]. In the most recent experience, the Spanish Head and Neck Cancer Cooperative Group Phase 2 study reported a high rate of 70% survival with functional larynx at 3 years with IC using TPF, followed by bioradiotherapy using cetuximab [17].

In contrast to the findings of this study, some studies have demonstrated better 2- and 5-year OS and EFS in the surgery arm compared to the non-surgical treatment arm [17, 18].

Considering the disadvantages and morbidity of TL [19, 20] and based on the above studies, recent updates to the American Society of Clinical Oncology guidelines have supported the use of organ preservation for selected patients with T3 or small T4a and a functional larynx [21].

At the time of the VALCSG study, around 25% of patients had T4 disease, while less than 10% had cartilage invasion [14]. In the more recent large trials like RTOG 91 − 11, T4 patients comprised only 10% of all patients due to the exclusion of full-thickness thyroid cartilage and base-of-tongue involvement [12]. In the GORTEC 2000-01 trial, T4 patients comprised only 13–15% of the study population [22]. In the TREMPLIN study, T4 was an exclusion criterion [16], but in the Spanish group study, 22% of the patients had T4 disease [17]. Thus, we should explore community and retrospective studies for the outcome of radiotherapy in T4 patients. Some studies showed better OS with surgery and ajuvant RT than definitive CRT in T4 cases [9, 23–26]. On the contrary, in a recently published study from Memorial Sloan Kettering Cancer Center, Eita et al. reviewed T4-stage cases from 1997 to 2015, analyzing the outcomes of 44 larynx and 53 hypopharynx cancer patients who received nonoperative management in whom cannot undergo surgery or choose not to have it. The 2- and 5-year OS rates for larynx cancer patients were 73% and 38%, while for hypopharynx cancer patients, they were 52% and 29% [27]Based on the above-mentioned studies, recent experiences and single high-volume center studies seem to have better outcomes than older or community based studies.

One indicator of the outcome in T4 patients is the reason for obviating surgery. The NCCN guidelines recommend larynx preservation only for those who decline surgery in the T4 stage [28]. In our study, the most common reasons for patients with T4 laryngeal cancer not undergoing surgery were patient refusal (46.7%) and selected T4a cases with functional larynx suitable for preservation (33.3%). These findings highlight the importance of patient preference and the potential benefits of laryngeal preservation for some patients with T4a disease. The outcomes of patients with T4 disease, which comprised 29% of our patients (about 24% in the definitive and 40% in the adjuvant groups), were inferior to those of the overall study population. We found around a 16% difference in 3-year DFS in favor of those who underwent surgery followed by adjuvant RT although with a modest difference in 3-year OS. The main reason for this remarkable difference in DFS is the rate of distant failure and not local control (Table 3). Although RT can halt the local growth of the tumor, the residual disease may get the chance to spread to other distant organs. The challenge that is not faced in patients who undergo surgery and removal of the entire larynx. Another explanation is the low rate of induction chemotherapy in our cohort (17%), considering the 36% rate of the N2-3 stage in the definitive group. We know that in patients with N2-3 HNSCC, induction chemotherapy raised the DFS rates in several clinical trials [29]. However, following the disease recurrence, the introduction of potent systemic therapy combinations like the TPextreme regimen has been able to control the disease and prolong overall survival [30].

One of the strengths of the present study was the evaluation of multiple outcomes, including OS, DFS, DMC, and LRC. We Included both definitive and adjuvant treatment options, allowing for a comparison of survival outcomes and providing data on patient preferences for treatment selection, which can be valuable for informed decision-making. Nevertheless, the present study had some limitations, as well. First, the retrospective nature of the study on a hospital-based population introduces potential selection bias, which may influence the results. Also, comparing patients with clinical stages in the definitive group vs. pathological stages in the adjuvant group has inherent biases. Additionally, the study did not assess the effect of treatment on other outcomes, such as adverse effects, toxicity, QOL, and laryngectomy-free survival. Our study was limited by its relatively small sample size. Additionally, the median follow-up of 29 months may not be long enough to capture all long-term survival outcomes; however was a reasonable timeframe to capture some long-term outcomes, particularly in T4 laryngeal cancer. Future studies with larger patient cohorts and more extended follow-up periods are needed to definitively compare the effectiveness of definitive and adjuvant RT particularly for T4 laryngeal cancer.

## Conclusions

This study suggests that both definitive and surgery followed by adjuvant radiotherapy may be reasonable treatment options for patients with T4 laryngeal cancer. The choice of treatment may depend on various factors, including patient preferences, tumor characteristics, and surgeon expertise. Considering the greater adverse effects of surgery, we suggest definitive chemoradiotherapy and reserving surgery as a backup plan for salvage treatment in these patients. Further research is needed to determine the long-term effectiveness of these treatments and to identify the optimal treatment approach for this patient population.

## Data Availability

Data is accessible upon a legitimate request to the corresponding author.

## Abbreviations

QOL: Quality of life
OS: Overall survival
RT: Radiotherapy
SCC: Squamous cell carcinoma
MRI: Magnetic resonance imaging
CT: Computed tomography
5FU: 5-Fluorouracil
ENE: Extranodal extension
GTV: Gross tumor volume
CTV: Clinical target volume
PTV: Planning target volume
DMFS: Distant metastasis control
RTOG: Radiation Therapy Oncology Group
